# Missing value imputation for microRNA expression data by using a GO-based similarity measure

**DOI:** 10.1186/s12859-015-0853-0

**Published:** 2016-01-11

**Authors:** Yang Yang, Zhuangdi Xu, Dandan Song

**Affiliations:** Department of Computer Science and Engineering, Shanghai Jiao Tong University, 800 Dongchuan Rd., Shanghai, 200240 China; Key Laboratory of Shanghai Education Commission for Intelligent Interaction and Cognitive Engineering, Shanghai, 200240 China; School of Computer Science and Technology, Beijing Institute of Technology, Beijing100081, China

## Abstract

**Background:**

Missing values are commonly present in microarray data profiles. Instead of discarding genes or samples with incomplete expression level, missing values need to be properly imputed for accurate data analysis. The imputation methods can be roughly categorized as expression level-based and domain knowledge-based. The first type of methods only rely on expression data without the help of external data sources, while the second type incorporates available domain knowledge into expression data to improve imputation accuracy.

In recent years, microRNA (miRNA) microarray has been largely developed and used for identifying miRNA biomarkers in complex human disease studies. Similar to mRNA profiles, miRNA expression profiles with missing values can be treated with the existing imputation methods. However, the domain knowledge-based methods are hard to be applied due to the lack of direct functional annotation for miRNAs. With the rapid accumulation of miRNA microarray data, it is increasingly needed to develop domain knowledge-based imputation algorithms specific to miRNA expression profiles to improve the quality of miRNA data analysis.

**Results:**

We connect miRNAs with domain knowledge of Gene Ontology (GO) via their target genes, and define miRNA functional similarity based on the semantic similarity of GO terms in GO graphs. A new measure combining miRNA functional similarity and expression similarity is used in the imputation of missing values. The new measure is tested on two miRNA microarray datasets from breast cancer research and achieves improved performance compared with the expression-based method on both datasets.

**Conclusions:**

The experimental results demonstrate that the biological domain knowledge can benefit the estimation of missing values in miRNA profiles as well as mRNA profiles. Especially, functional similarity defined by GO terms annotated for the target genes of miRNAs can be useful complementary information for the expression-based method to improve the imputation accuracy of miRNA array data. Our method and data are available to the public upon request.

## Background

Missing values are commonly present in microarray data due to various reasons, such as the limitation on detection sensitivity, contamination or error induced in experimental operations, and inappropriate data preprocessing. For the sake of accurate analysis on expression profiles, the estimation of missing values has been a basic and key issue in microarray data analysis.

Up to now, a variety of methods have been proposed for imputing missing values in gene expression profiles, which mainly fall into two categories. The first type is based solely on expression data, such as KNNImpute [1], LLSimpute [2] and Bayesian method [3]. The second type is based on domain knowledge in addition to expression data [4, 5].

*K*-nearest neighbor (*K*NN) is the most widely used algorithm for missing value imputation, and many other methods implement various modified versions of *K*NN process. For example, assume that gene *g*_1_ whose value on the first sample is missing, then the Euclidean distances of expression levels between *g*_1_ and other genes are measured as Eq. 1.

(1) $$ d_{g_{1},g_{j}} = \sqrt{\frac{\sum\limits_{i={s_{1}}}^{s_{n}}(G_{1,i}-G_{j,i})^{2}}{n}},  $$

where $d_{g_{1},g_{j}}$ denotes the distance between *g*_1_ and *g*_*j*_. *G*_1,*i*_ and *G*_*j,i*_ are the expression values of *g*_1_ and *g*_*j*_ at the *i*th sample, respectively. {*s*_1_,⋯,*s*_*n*_} are the indexes of *n* samples where both *g*_1_ and *g*_*j*_ have valid values. It should be noted that *g*_1_ and *g*_*j*_ may have more than one missing values at different samples, and only the samples that both genes have valid values are considered. Thus the squared differences of expression values are averaged over *n*, i.e. the number of samples considered.

Once the distances between *g*_1_ and all other genes have been computed, *K* nearest genes are selected and a weighted average of their expression values on the first sample is assigned to *G*_1,1_.

In many cases, gene expression level alone cannot guarantee good imputation result because of noise, bias or a large proportion of missing values. Actually, the microarray experimental results are not simply numerical matrices. Instead, these expression data should be treated with the consideration of biological background, such as functional similarity, biological pathways that genes involved and interactions between gene products. If the external annotation data sources can be effectively incorporated into the algorithms, the imputation quality would be improved.

Common annotation data include Gene Ontology (GO) [6], KEGG pathways [7], and GenMAPP [8] etc. Since GO annotation covers most of human gene products, GO is most widely used for measuring functional similarity between genes. GO provides a controlled and standardized vocabulary, which has become an important tool and knowledge base in bioinformatics research. GO consists of three organizing principles, i.e. cellular component, biological process and molecular function. It represents gene characteristics of the three categories in terms, which are the basic elements of the GO database organized in directed acyclic graphs (DAG) including nodes (terms) and relationships (edges). The semantic similarity between GO terms regarding the locations in the DAG and common ancesters/descendands directly reflects the association between gene products [9, 10].

In the past two decades, many efforts have been put on studying the semantic similarity of GO terms. Early methods refer to related studies in natural language [11–13], and mainly consider information content of GO terms, as defined in Eq. 2. The drawback of these methods is that they care little about structural information, and treat the GO structure as a tree instead of a DAG.

(2) $$ IC(x)=-\log p(x)=-\log\left(\frac{|\textit{G}_{x}|}{|\textit{G}_{root}|}\right),  $$

where *p*(*x*) is the probability of an attribute described by a GO term *x*, *G*_*x*_ is the set of genes that are associated with GO term *x* (i.e. genes annotated by *x* and all of their descendants), and *G*_*root*_ is the set of genes that are associated with the root term (i.e. all genes that the DAG can annotate). Here, we use ‘associated with’ instead of ‘annotated with’ because in GO DAGs, parent nodes are generalized concept of children nodes, thus a gene annotated with some terms is also associated with their parent nodes. Recent methods [14–17] consider more structural information, such as G-SESAME [15] (http://bioinformatics.clemson.edu/G-SESAME/), which considers both common ancestors of two GO terms and their relative location in the GO graph.

Tuikkala et al. [4] investigated whether semantic similarity could improve the performance of missing value imputation. Their experimental results demonstrated that even a small proportion of annotated genes can provide improvements in data quality helpful for imputation. Besides GO, other biological knowledge has also been applied to missing value imputation. Xiang et al. [18] proposed a histone acetylation information aided imputation method, called HAIimpute, which incorporates histone acetylation information into conventional *K*NN and LLS algorithms. Ni et al. used protein-protein interaction (PPI) annotation data, and proposed PPI-KNNimpute and PPI-LLSimpute methods [5]. All of these three studies tested their methods on yeast cDNA microarray data, benefitting by the comprehensive annotation available for yeast.

In recent years, miRNA microarrays have become more and more common because miRNAs play important roles in many biological processes and the development of complex diseases. Especially, the correlation of miRNA expression profiles with clinical-pathological characteristics of cancer patients has been largely investigated. MiRNA expression data is of great help for tumor classification and can be effective biomarkers for prognosis even more precisely than protein-coding genes [19, 20]. The miRNAs expression profiles also have missing values due to the low expression intensity of miRNAs. Basically, the estimation of missing values in miRNA arrays can follow the same strategies for DNA arrays. However, the domain knowledge is hard to be utilized because there is no direct GO annotation or similar standardized functional annotation for miRNAs. Therefore, the imputation of missing values for miRNA arrays with domain knowledge is a relatively tough job and seldom studied.

Considering that miRNAs play their roles mainly by regulating the expression of target genes at post-transcription level, the functional similarity of miRNAs can be inferred based on GO semantic similarities of their target genes [21]. Identification of target gene is an important issue and also a well-studied bioinformatics task in miRNA study. Some miRNA databases record known targets, such as TarBase [22], which collects manually curated target genes with experimental support. Widely used computational tools for predicting targets of mammalian miRNAs include picTar [23], TargetScan [24], miRanda [25, 26], etc. The GO annotation of target genes can be searched in GO database and pairwise similarity can be defined. Then the functional similarity between miRNAs can be computed according to their target genes’ similarities.

We combine miRNA functional similarity with Euclidean distance of expression profile and develop a new imputation method. The new method is evaluated on two miRNA datasets from breast cancer research, on which the new method achieves improved performance compared with the expression-based method. The experimental results demonstrate that the functional knowledge retrieved from target genes of miRNAs can help to improve the estimation of missing values for miRNA expression profiles.

## Results and discussion

### Data source

In this study, we used two public miRNA data sets from NCBI [27], namely GSE26659 [28] and GSE40525 [29]. Both profiles were measured by Agilent-019118 Human miRNA Microarray 2.0 G4470B containing probes for 723 human and 76 human viral microRNAs from the Sanger database v.10.1.

In order to evaluate the performance of the new method, the original data sets were preprocessed by removing miRNAs with missing values. Then new data sets with different percentages of missing values were simulated from the preprocessed complete data. Considering that missing values are generally not evenly distributed over the whole profile, we did not generate the data sets by randomly selecting missing elements. Instead, we randomly selected 50 % of the miRNAs which were assumed to have incomplete profiles, and then randomly assigned missing elements in each of these miRNAs’ profiles. The missing rate was set to be a random integer percentage between 1 and *p* % (maximum missing rate). Obviously, the bigger *p* is, the more missing values are present in the generated data sets. When *p* is equal to 20, it can be calculated that the expected value of missing rate of the whole simulated data set is 5 %. By changing the value of *p*, we can get simulated sets with different missing rates. The generation of data sets was repeated 100 times. Different imputation methods were tested and evaluated on all the simulated data set, and the accuracies were averaged to obtain the final result.

### Evaluation criteria

There are multiple criteria for evaluating imputation algorithms. A commonly used one is the normalized root mean square error (*NRMSE*). Let *G*_*est*_ be the estimated data matrix by the imputation algorithm, and *G*_*true*_ be the matrix of the original complete data matrix. The *NRMSE* is defined as:

(3) $$ NRMSE=\frac{\sqrt{mean[(G_{est}-G_{true})^{2}]}}{\sigma[G_{true}]},  $$

where *σ* denotes standard deviation.

### Experimental results

**Fig. 1:**
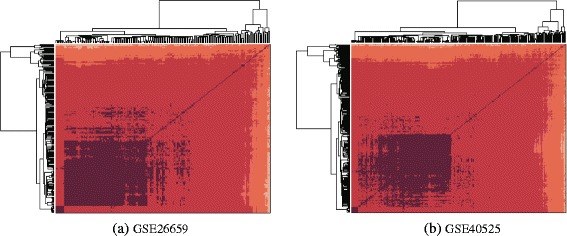
Hierarchical clustering of functional similarity matrix of miRNAs

**Fig. 2:**
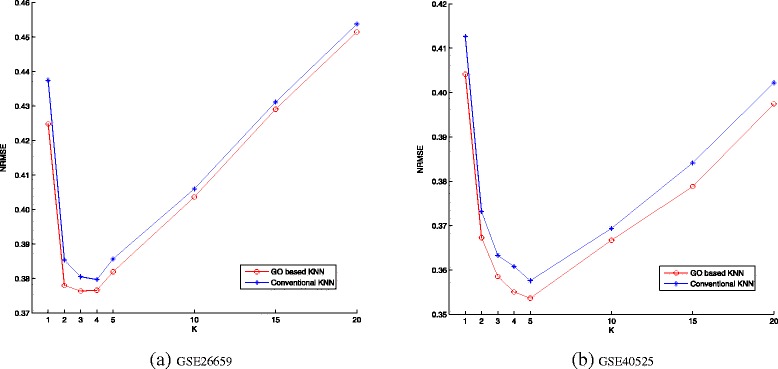
Comparison of NRMSE at different values of *K*

Based on semantic similarity retrieved from GO graph and BMA (best match average) strategy for calculating similarity of two sets, we obtain the GO-based functional similarity matrix *H* of miRNAs. Before proceeding to the evaluation of the new method, we conduct a hierarchical clustering and draw a heat map of *H*. Figure 1(a) and (b) show clustering results for the data sets extracted from GSE26659 and GSE40525, respectively. It can be observed that the miRNAs are grouped into four large clusters distinguished by depths of color. Different from conventional imputation method, given the new similarity measure, the nearest neighbors of a certain miRNA are more likely chosen within its function cluster. We use the new similarity measure in *K*NN imputation method. Figure 2(a) and (b) compare the *NRMSE* of conventional *K*NN and GO-based *K*NN with different values of *K* for GSE26659 and GSE40525, respectively, where maximum missing rate is 40 % (the expected value of missing rate for the whole simulated data set is 10 %). GO-based *K*NN has better performance than conventional *K*NN no matter what the value of *K* is. The results also suggest that *K* has great impact on the accuracy of imputation. The two data sets have a similar pattern of varying accuracies, and the optimal value of *K* is 4 or 5, which is much smaller than that used in the imputation for mRNA expression profiles.

**Fig. 3:**
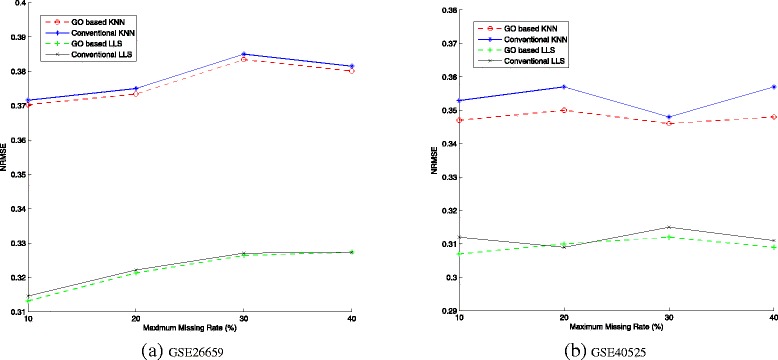
Comparison of NRMSE at different maximum missing rates

Figure 3(a) and (b) compare expression-based and GO-based methods at different maximum missing rates, by using *K*NN and LLS (local least squares). The maximum missing rate, i.e. *p*, is an upper bound of the percentage of missing values. For example, when *p* equals 40 %, the missing rate is randomly assigned between 1 and 40 %. In GSE40525 data set which has 120 samples, a miRNA which is selected to have missing values can have 48 missing values at most and one missing value at least with equal probability. Such generation of missing values is more practical than completely random model with regard to the real distribution in microarray data. Here *K* is set to be 5. Solid lines denote conventional methods, and dotted lines denotes GO-based methods. These two figures show that incorporating GO knowledge can improve the imputation accuracy for both *K*NN and LLS. The improvement in LLS is not as obvious as in *K*NN, since we only use GO-based measure to select neighbors in LLS, thus the GO information has less impact on the performance of LLS than that of *K*NN. Generally, the LLS method has much less *NRMSE* while takes more computation time compared with *K*NN.

Compared with previous studies which utilize GO information for the imputation of missing values on mRNA expression data, the improvement on miRNA data is not that significant. Actually, we have found that the *NRMSE* of miRNA data sets using conventional imputation methods is much less than that of mRNA data. The possible reasons are: a) miRNA profiles with hundreds of miRNA have much smaller scale than mRNA profile with tens of thousands of probes; b) the expression levels of the same miRNA vary slightly across samples. In addition, we observe that higher maximum missing rate does not necessarily results in bigger *NRMSE*. Similar cases are also present in mRNA expression data sets [4]. And the non-completely random generation of missing values may also increase the probability of such result.

## Discussions

This study focuses on effective incorporation of biological knowledge into the imputation of missing values in miRNA microarray data. Unlike protein-coding genes, the functional annotation of miRNAs cannot be utilized directly because no such database is available. Since miRNAs play their roles mainly by suppressing or silencing their target genes’ expression, two miRNAs may have strong correlation on their function and expession pattern if their targets are involved in the same biological process or have similar function. By transferring GO-based pairwise similarity of genes to the similarity of two target sets, the functional similarity of two miRNAs can be indirectly inferred.

Although the experiments have demonstrated the performance improvement, it is obvious that biological knowledge can only be treated as a data source complementary to expression levels. On one hand, function similarity does not directly correlate with expression similarity [30]. Similar function only indicates that two miRNAs have similar expression pattern with high probability. On the other hand, the process of calculating GO-based similarity for miRNAs has some drawbacks.

Firstly, the GO-based semantic similarity does not accurately reflect gene function similarity. Although we retrieve semantic information in the Molecular Function DAG, and the G-SESAME method has considered structural information, there is still a lot of information lost here. For example, the method only considers common ancestors but neglects common descendants. Moreover, even though we have a perfect method which comprehensively utilizes GO graph information, this semantic similarity is not necessarily corresponds to real functional similarity because the annotation is incomplete.

Secondly, in order to cover as many miRNAs in our data set as possible, we adopt computational tools for target gene identification, which may have unreliable results although we have set a threshold to control false positives and false negatives.

In addition, some process steps within the whole workflow, which have great impact on the final results, need further studies. The first issue is the simulation strategy for generating the evaluation set with artificial missing values. Although the partial-random model used in this study seems more reasonable than complete random model, how to simulate missing values according to their real distribution in microarray profiles needs careful studies. The second issue is the integration strategy from pair-wise GO similarity to miRNA similarity. Here a two-step best match average method is conducted, while other strategies may be examined. For example, each miRNA can be directly regarded as a GO set with redundant GO terms from its targets. A pairwise similarity for such redundant set can be defined.

As more and more miRNA functions have been revealed, available annotation for miRNAs increase rapidly, and other biological knowledge with regard to miRNAs can also be utilized. For example, Wang et al. [30] used microRNA-associated diseases to define miRNA functional similarity. Undoubtedly, the miRNA functional similarity provides complementary but important information to ensure the quality of data analysis on miRNA expression profiles.

## Conclusions

Biological domain knowledge has been demonstrated to be very helpful to improve the imputation of missing values in DNA microarray data profiles. However, because of the lack of function annotation for miRNAs, the domain knowledge-based imputation methods have rarely been studied for miRNA expression data. In this paper, we propose to use the pairwise functional similarity of miRNAs based on the semantic similarity of target genes in GO graph as a complementary information for expression data and define a new similarity measure. This new measure can work together with conventional *K*NN and other imputation methods. The experiments have been conducted on two breast cancer miRNA expression profiles, and the normalized root mean square error (*NRMSE*) is compared under different values of *K* and maximum missing values. The GO-based new imputation method shows decreased *NRMSE* on both data sets.

Our method is applicable to not only human miRNAs but also other species as long as the target genes can be obtained. With the rapid accumulation of miRNA microarray data, the studies on domain knowledge-based function similarity for miRNAs are of great need to improve expression data quality and eventually benefit for the identification of novel miRNA biomarkers and novel potential functions of miRNAs.

## Methods

As shown in Fig. 4, our method consists of four major parts. The first part builds a connection between miRNAs and target genes. The second part builds another connection between genes and GO terms. The third part focuses on the computation of pairwise similarity of GO terms, and the last part implements *K*NN (or other imputation algorithm) with the new similarity measure. The whole workflow can be summarized in the following steps:

1. Identify target genes of miRNAs via computational prediction tools or public databases

**Fig. 4:**
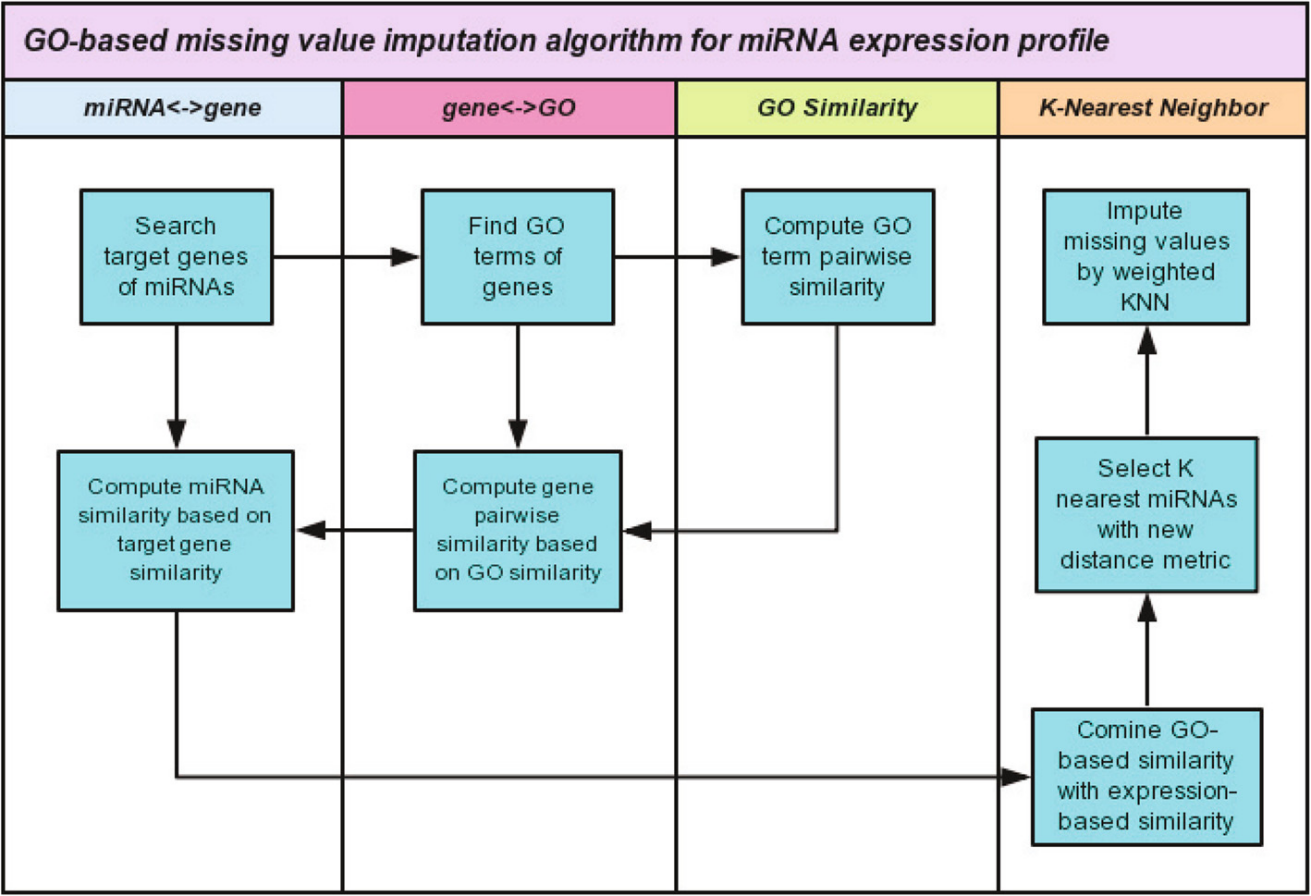
Flowchart of GO-based imputation algorithm for miRNA expression data
2. Search GO terms annotated for each target gene obtained in Step 1
3. Compute similarity for each pair of GO terms obtained in Step 2
4. Compute similarity for each pair of target genes from the similarity of their GO terms
5. Compute similarity of each pair of miRNAs from the similairty of their target genes
6. Define new similarity measure by combining GO-based similarity and expression level-based distance
7. Impute missing values with the new measure

### GO similarity

In order to compute the functional similarity of two genes *g*_1_ and *g*_2_ from their GO annotation, we firstly retrieve GO term list of each target gene for miRNAs. Two genes correspond to two GO sets, i.e., $T_{g_{1}}=\{t_{1},t_{2},\cdots,t_{k}\}$, $T_{g_{2}}=\{t_{1}',t_{2}',\cdots, t_{s}'\}$, and the computation of gene pairwise similarity turns out to be the similarity between two GO sets. There are two issues here. The first issue is to define the similarity between two GO terms according to their correlation in the GO DAGs. We adopt G-SESAME implemented in R package GOSemSim [31], and compute GO similarity in the DAG of Molecular Function (MF), which is a widely used knowledge base of gene function. The second issue is how to integrate the pairwise similarities to the similarity between two sets.

Given two sets of GO terms $T_{g_{1}}$ and $T_{g_{2}}$, which have *k* and *s* terms respectively, and similarity of each pair of GO terms, there are four basic methods to obtain gene similarity, i.e. the similarity of these two sets, namely *max*, *avg*, *rcmax* and *BMA* [31]. The former two methods compute similarity for each pair of (*t*_1_,*t*_2_), where $\phantom {\dot {i}\!}t_{1} \in T_{g_{1}}$ and $\phantom {\dot {i}\!}t_{2} \in T_{g_{2}}$, and use the maximum (max) or average (avg) value to represent the similarity of the two genes. These two methods have obvious disadvantages. The *max* strategy fails to consider the overall similarity of two sets. For example, it regards the similarity of two sets as 1 even if they have only one term in common. As for the *ave* strategy, even though the two sets are exactly the same, the similarity between two sets is not 1. The *rcmax* method works like following:

1. For each term $\phantom {\dot {i}\!}t_{i} \in T_{g_{1}}$, find the maximum similarity between *t*_*i*_ and the terms in $T_{g_{2}}\phantom {\dot {i}\!}$
2. Get average value of the *k* maximum values obtained for all terms in $T_{g_{1}}$ from 1), and name it as *max*_1_
3. For each term $\phantom {\dot {i}\!}t_{j}\in T_{g_{2}}$, find the maximum similarity between *t*_*j*_ and the terms in $T_{g_{1}}\phantom {\dot {i}\!}$
4. Get average value of the *s* maximum values obtained for all terms in $T_{g_{2}}$ from 3), and name it as *max*_2_
5. Assign *max*(*max*_1_,*max*_2_) to the gene similarity

*rcmax* is often biased when the sizes of GO sets differ considerably. The last method *BMA* (best match average) has the same steps 1) and 3) of rcmax. The difference is that it calculates the overall average value of *k* maximum values of terms in $T_{g_{1}}$ and *s* maximum values of terms in $T_{g_{2}}$. The gene similarity is defined in Eq. 4.

(4) $$ {} sim_{BMA}(g_{1},g_{2})=\frac{\sum\limits_{i=1}^{k} \max\limits_{1\leq j\leq s} sim(t_{i},t_{j}')+\sum\limits_{j=1}^{s} \max\limits_{1\leq i\leq k} sim(t_{i},t_{j}')}{k+s}  $$

*BMA* has been used in many studies [15, 21]. Here we also adopt *BMA* to compute gene similarity based on GO pairwise similarity. Since each miRNA corresponds to a set of target genes, miRNA similarity can be computed by gene similarity in the same manner.

In this study, we use computational prediction tools to identify targets of miRNAs, because the experimental-supported target databases, like TarBase, only house a small portion of miRNAs in the data sets. We have found that miRanda provides prediction results for the most miRNAs in our date sets than other tools. In order to avoid too many false positives, we set a threshold of mirSVR score ≤−1 to ensure the prediction accuracy. It should be noted that the naming of some miRNAs is inconsistent between databases and miRNA array. We refer to miRBase [32] to deal with the inconsistency.

### GO-based imputation algorithm

In our method, we combine the GO-based functional similarity with the Euclidean distance obtained from expression profiles as a new measure. Here we adopt a similar strategy used in Tuikkala’s work [4]. Given the functional similarity matrix *H* and Euclidean distance matrix *D*, each element of the new distance matrix *C* is defined as Eq. 5.

(5) $$ C_{i,j}=(1-H_{i,j})^{\alpha} D_{i,j},  $$

where *i,j*=1,⋯,*n*, and *n* is the number of miRNAs in the data set. *α* is a positive parameter of weight, denoting the impact of GO-based similarity in the combined measure. If *α*=0, *C* turns out to be *D*. The bigger *α* is, the more impact that GO information has on the selection of nearest neighbor and performance of imputation. The value of *α* is determined on an evaluation set extracted from the original complete data set.

Given the new distance matrix *C*, the following steps are the same as conventional expression-based imputation algorithm. Assume that we want to estimate miRNA *g*_*i*_’s missing value at the *t*th sample, and the nearest *K* miRNAs according to the new measure are $g_{s_{1}},\cdots, g_{s_{K}}$. The estimated value $\bar {G}_{i,t} = w_{s_{1}}G_{s_{1},t} + w_{s_{2}}G_{s_{2},t} + \cdots + w_{s_{K}}G_{s_{K},t}$. For *K*NN, $w_{s_{j}}=\frac {1/C_{g_{i},g_{s_{j}}}}{\sum 1/C_{g_{i},g_{s_{j}}}}, 1\leq j \leq K$, and for LLS, $w_{s_{j}}$ are the coefficients of the linear combination, obtained by solving the least squares optimization problem.
